# Data set of climatic factors measured in a low latitude region with warm and humid climate: Solar radiation, cloud cover and sky temperature

**DOI:** 10.1016/j.dib.2021.107404

**Published:** 2021-09-20

**Authors:** Jefferson Torres-Quezada, Helena Coch, Antonio Isalgué

**Affiliations:** a School of Architecture, Engineering, Industry and Construction Unit, Catholic University of Cuenca, Ave. Americas & General Torres Engineering building, 1st floor, Cuenca-Ecuador 010102, Ecuador; b Architecture, Energy and Environment, School of Architecture, Polytechnic University of Catalonia, Spain; c Physics Department, Polytechnic University of Catalonia, Spain

**Keywords:** Warm-humid climate, Solar radiation, Sky temperature, Cloudiness

## Abstract

The data contained in this article refers to the measurements of climatic factors during an entire year in low latitudes regions with warm and humid climates, in Santa Rosa city from Ecuador. Since the geographical location and the importance of cloudiness in these regions, these measurements have focused on the solar radiation flux, cloud cover and sky temperature. The data obtained have been configured in a weather file for energy calculations, which has been used in the research article “Assessment of the reflectivity and emissivity impact on light metal roofs thermal behaviour, in warm and humid climate” [1].

## Specifications Table

**Table:** 

Subject	Architecture
Specific subject area	Building Thermal Behaviour
Type of data	Table Figure
How data were acquired	Data was collected from measurements in situ, using: Meteorological station: Davis Vantage Pro2 Plus (Wireless) Infrared Thermometer Testo 830-t4 Visual Observation in situ
Data format	Raw Analysed
Parameters for data collection	Climatic factors measured throughout the year 2016. Cloud cover and sky temperature measured in October and December.
Description of data collection	Solar radiation, temperature, humidity, air velocity, wind direction measurements were taken in intervals of 10 min. The sky measurements were taken in intervals of 2 hours, daytime and night time.
Data source location	City/Town/Region: Santa Rosa/El Oro/Ecuador
Data accessibility	With the article
Related research article	J.Torres-Quezada, H. Coch & A. Isalgué, Assessment of the reflectivity and emissivity impact on light metal roofs thermal behaviour, in warm and humid climate. Energy and Buildings. 188-189 (2019) 200-2008. https://doi.org/10.1016/j.enbuild.2019.02.022

## Value of the Data

• The data shown in this article contributes to the solar radiation data from the Coast of Ecuador obtained by measurements, which until now has been approximated only by calculations.
• The provided information could be used to analyse the different components of the solar radiation in this region: direct and diffuse. In the same way, this information contributes to the determination of radiative cooling capacity of the sky at these latitudes.
• The weather file included in this article, which is based on measurements *in situ*, can be used for different energetic software, and, hence for an accurate thermal analysis of buildings in these regions.

## 1 Data Description

The data collection process used in this article had the purpose to established the analysis days conditions of the related research article entitled “Assessment of the reflectivity and emissivity impact on light metal roofs thermal behaviour, in warm and humid climate” [1], specifically an average day and an extreme day. For this, an annual preliminary evaluation has been carried out based on historical data. From here two months have been chosen with the average climatic conditions sought, October and December. Finally, several weeks of these two months have been measured, where the parameters of the analysis days have been found in the two weeks shown in this article (average day: October 12 and December 7, extreme day: October 10 and December 4).

**Fig. 1:**
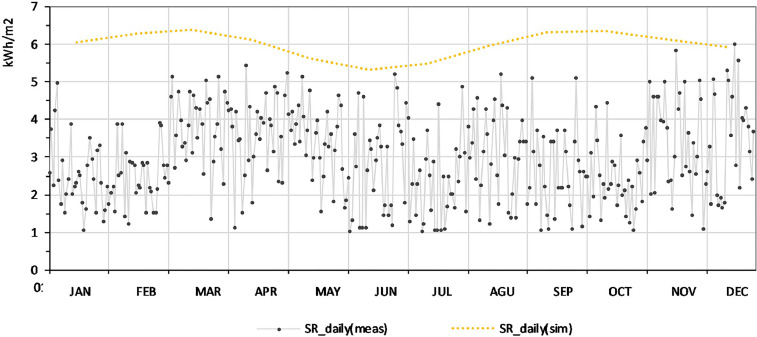
Daily solar irradiation: measured by meteorological station [2] and simulated with a clear sky conditions with the use of Heliodon software [3].

The climatic parameters measured were collected from a meteorological station throughout the whole year of 2016 [2]. Fig. 1 shows the daily average of the solar irradiation on the horizontal plane throughout the measured year. In addition, this figure shows this parameter obtained by simulations with the use of the Heliodon software [3], which considers a day with clear sky conditions at this latitude. All measured climatic factors are shown in Supplementary Table 1.

In addition to these measurements, hourly data of solar irradiation, cloud cover, air temperature and sky temperature were measured *in situ* during several weeks in October and in December. As mentioned before, these two months contain days with different climate conditions, some of them coincide with the values established for model day with average and extreme conditions. So, one week from every month, which contains these days, are shown below.

**Fig. 2:**
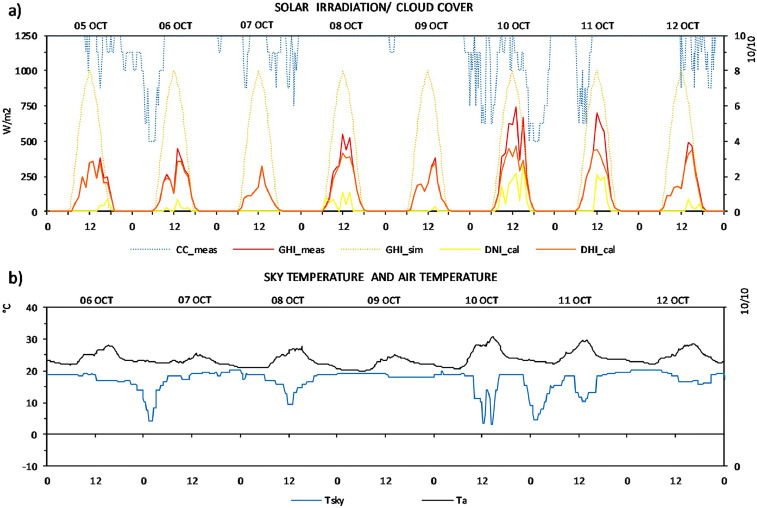
Data of October: (a) measured cloud cover (CC_meas), measured global horizontal irradiation (GHI_meas), simulated horizontal solar irradiation (GHI_sim), calculated direct normal (DNI_calc) and diffuse horizontal (DHI_calc) irradiation, b) measured air temperature (Ta) and measured sky temperature (Tsky).

**Fig. 3:**
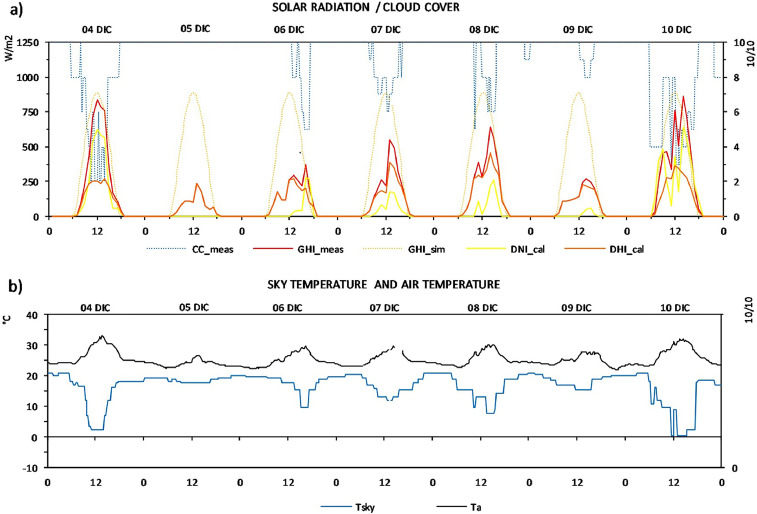
Data of December: (a) measured cloud cover (CC_meas), measured global horizontal irradiation (GHI_meas), simulated horizontal solar irradiation (GHI_sim), calculated direct normal (DNI_calc) and diffuse horizontal (DHI_calc) irradiation, (b) measured air temperature (Ta) and sky temperature (Tsky).

Fig. 2 shows the data of measured cloud cover, measured global horizontal irradiation, simulated solar irradiation with clear sky conditions, calculated direct normal and diffuse horizontal irradiation based on measurements, and, Fig. 3 contains the measured data of sky temperature and air temperature; in 8 and 7 days respectively of these two months. Since the high cloudiness in this region, the measurements of these two last measured parameters were intended to give an approximation more accurate of the thermal behavior of the sky and its radiative cooling effect. These measured data are also shown in Supplementary Table 2.

Finally, a weather file was configured based on the measurements made in October, which is shown in Supplementary Table 3. The climatic factors shown in this file are: air temperature, dew point, humidity, horizontal infrared radiation, global solar radiation, normal direct solar radiation, diffuse solar radiation, wind velocity, wind direction, cloud cover, rain fall, etc.

Although this weather file is only one week of the year, the days shown have different climatic conditions relevant for the climate of this region, like the average day (October 12th) or the extreme day (October 10th).

## 2 Experimental Design, Materials and Methods

Regarding solar radiation, air temperature, humidity, dew point, rainfall, air velocity and wind direction, were collected in intervals of 10 min from a meteorological station with the following characteristics throughout 2016.

**Table:** 

METEOROLOGICAL STATION SPECIFICATIONS	

Hardware:	Davis Vantage Pro2 Plus
Software:	Davis Vantage Pro2 Plus (Wireless)
Coordinates:	3°26′39″S, 79°59′38″O
Elevation:	20m

SOLAR RADIATION	

Device:	Solar radiation sensor: Apogee Pyranometer SP-110
Calibration:	5000W/m2 per V_manufacturer calibration
Resolution:	1 W/m2
Range:	0-1800 W/m2
Accuracy:	5%
Cosine Response:	+/‒ 3% for angles 0-75°
Uncertainties:	+/‒ 1%

AIR TEMPERATURE	

Device:	Integrated Sensor Suite (ISS) in Davis Vantage Pro2 Plus
Calibration:	manufacturer calibration
Resolution:	0.1 °C
Range:	‒40 °C-65 °C
Accuracy:	0.5 °C
Uncertainties:	+/‒ 0.5 °C

HUMIDITY	

Device:	Integrated Sensor Suite (ISS) in Davis Vantage Pro2 Plus
Calibration:	manufacturer calibration
Resolution:	1%
Range:	1–100%
Accuracy:	3–4% above 90%
Uncertainties:	3–4%

DEW POINT	

Device:	Integrated Sensor Suite(ISS)/Hum Station in Davis Vantage Pro2 Plus
Calibration:	manufacturer calibration
Resolution:	0.1 °C
Range:	−76 °C to + 54 °C
Accuracy:	1.5 °C
Uncertainties:	+/− 1.5 °C

RAINFALL	

Device:	Tipping-bucket rain collector in ISS in Davis Vantage Pro2 Plus
Calibration:	0.01″ rainfall increment or 0.2 mm _ manufacturer calibration
Resolution:	0.2 mm
Range:	0.2 mm to 999.8 mm (daily)
Accuracy:	4%
Uncertainties:	+/− 1 mm (rain rate)

AIR VELOCITY	

Device:	Anemometer, Integrated Sensor Suite in Davis Vantage Pro2 Plus
Calibration:	manufacturer calibration
Resolution:	1 km/h
Range:	3–322 km/h
Accuracy:	3 km/h
Uncertainties:	+/− 5%

WIND DIRECTION	

Device:	Anemometer, Integrated Sensor Suite in Davis Vantage Pro2 Plus
Calibration:	manufacturer calibration
Resolution:	1°
Range:	0–360°
Accuracy:	3°
Uncertainties:	+/−5%

These technical specifications are detailed in [4]

In regards to the additional measurements: cloud cover and sky temperature, these data were collected in intervals of 2 hours. The graphical changes shown in Figs. 2 and 3 about these parameters are conditioned by the time interval used in measuring it.

**Fig. 4:**
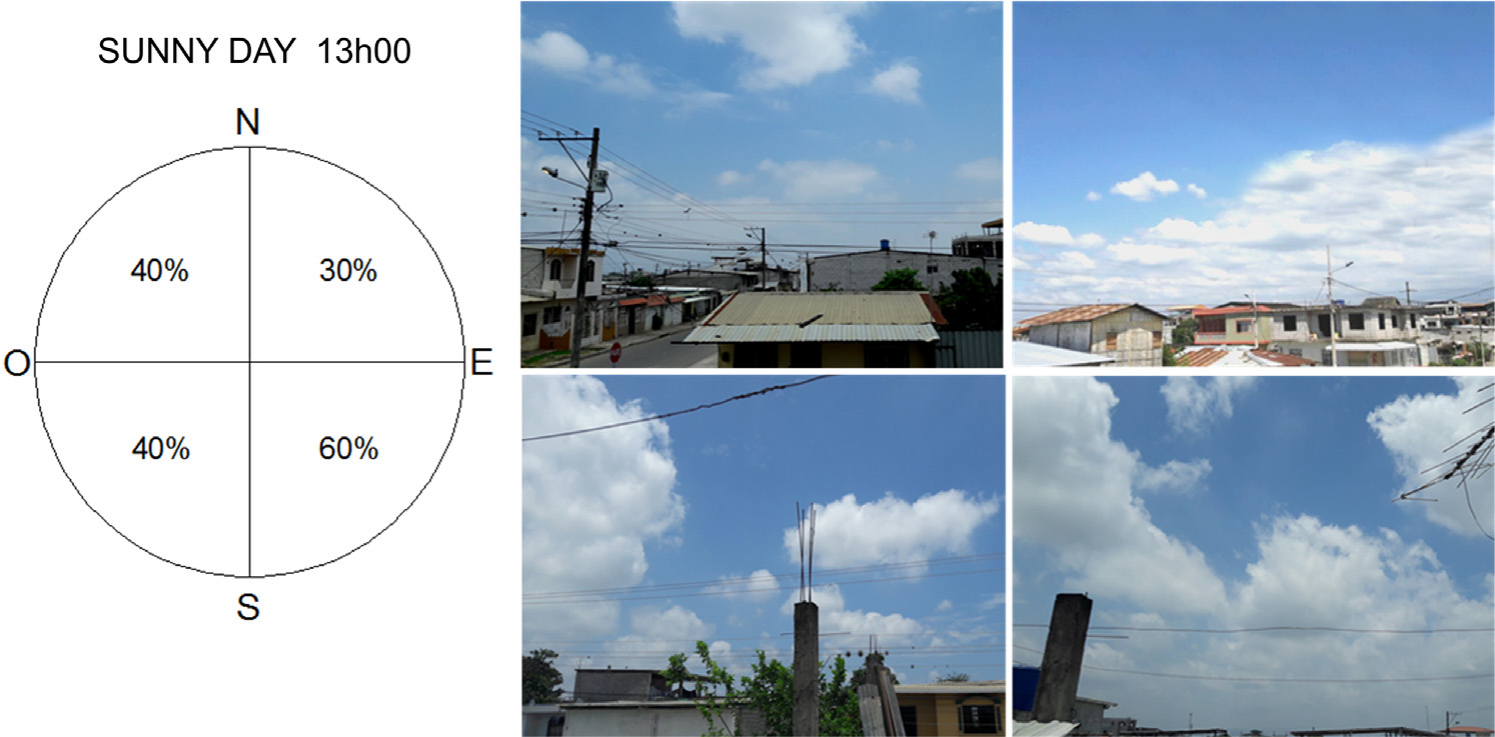
Process of cloud cover measurements: division of the sky vault in equal quadrants (left) and images of each quadrant in October 10th at 13h00 (right).

The process carried out to measure the cloud cover parameter was visual observation. This process consists in dividing the sky vault into equal quadrants, then, an approximation of percentage of the sky of each quadrant was made. By the last, the four percentages obtained were averaged. The unit used for the information collected was the tenths. This process was supported with a photographic base made simultaneously, see Fig. 4.

With respect to sky temperature measurements, due to the fact that the sky is not isotropic, its thermal behavior was described through the measurements of its temperature in different points of the sky vault. These measurements were taken from the skyline to the zenith, and they were averaged to obtain one sky temperature, which is the value shown in this article (*Tsky*). The equipment used for these measurements was an infrared thermometer TESTO 830 T4 with the following technical specifications.

**Table:** 

Device:	Non-contact Infrared Thermometer 830 T4
Measurement:	2 pointing lasers, 30:1 lens
Calibration:	emissivity 0.90
Resolution:	0.1 °C
Range:	−30 to + 400 °C
Accuracy:	1%
Emissivity:	0.1–1.0 (Adjustable)
Spectral range:	8–14 um
Uncertainties:	+/‒ 1.5 °C (‒20 to 0 °C); +/‒ 2 °C (‒20 to 0 °C); +/‒ 1 °C of m.v.(remaining range)

These technical specifications are detailed in [5]

## Ethics Statement

The work does not involve the subject of humans, animals, or data from social media platforms.

## CRediT authorship contribution statement

**Jefferson Torres-Quezada:** Conceptualization, Methodology, Investigation. **Helena Coch:** Writing – original draft, Supervision. **Antonio Isalgué:** Writing – original draft, Supervision.

## Declaration of Competing Interest

The authors declare that they have no known competing financial interests or personal relationships which have or could be perceived to have influenced the work reported in this article.

## Appendix Supplementary materials

Image, application 1

Image, application 2

Image, application 3
